# Rho-kinase inhibitor hydroxyfasudil protects against HIV-1 Tat-induced dysfunction of tight junction and neprilysin/Aβ transfer receptor expression in mouse brain microvessels

**DOI:** 10.1007/s11010-021-04056-x

**Published:** 2021-02-06

**Authors:** Qiangtang Chen, Yu Wu, Yachun Yu, Junxiang Wei, Wen Huang

**Affiliations:** 1 Department of Neurology, The First Affiliated Hospital, Guangxi Medical University, #6 Shuangyong Road, Nanning, 530021 Guangxi China; 2 Department of Neurology, The First People’s Hospital of Qinzhou, Qinzhou, 535099 Guangxi China

**Keywords:** HIV-1 transactivator protein, Rho/ROCK signaling pathway, Tight junction protein, Neprilysin, Receptor for advanced glycation end products, Low-density lipoprotein receptor-related protein 1

## Abstract

HIV-1 transactivator protein (Tat) induces tight junction (TJ) dysfunction and amyloid-beta (Aβ) clearance dysfunction, contributing to the development and progression of HIV-1-associated neurocognitive disorder (HAND). The Rho/ROCK signaling pathway has protective effects on neurodegenerative disease. However, the underlying mechanisms of whether Rho/ROCK protects against HIV-1 Tat-caused dysfunction of TJ and neprilysin (NEP)/Aβ transfer receptor expression have not been elucidated. C57BL/6 mice were administered sterile saline (i.p., 100 μL) or Rho-kinase inhibitor hydroxyfasudil (HF) (i.p., 10 mg/kg) or HIV-1 Tat (i.v., 100 μg/kg) or HF 30 min before being exposed to HIV-1 Tat once a day for seven consecutive days. Evans Blue (EB) leakage was detected via spectrophotometer and brain slides in mouse brains. The protein and mRNA levels of zonula occludens-1 (ZO-1), occludin, NEP, receptor for advanced glycation end products (RAGE), and low-density lipoprotein receptor-related protein 1 (LRP1) in mouse brain microvessels were, respectively, analyzed by Western blotting and quantitative real-time polymerase chain reaction (qRT-PCR) analyses. Exposure of the mice to HIV-1 Tat increased the amount of EB leakage, EB fluorescence intensity, blood–brain barrier (BBB) permeability, as well as the RAGE protein and mRNA levels, and decreased the protein and mRNA levels of ZO-1, occludin, NEP, and LRP1 in mouse brain microvessels. However, these effects were weakened by Rho-kinase inhibitor HF. Taken together, these results provide information that the Rho/ROCK signaling pathway is involved in HIV-1 Tat-induced dysfunction of TJ and NEP/Aβ transfer receptor expression in the C57BL/6 mouse brain. These findings shed some light on potentiality of inhibiting Rho/Rock signaling pathway in handling HAND.

## Introduction

Highly active antiretroviral therapy (HAART) is not capable of eradicating human immunodeficiency virus (HIV) infection in the central nervous system (CNS), although it succeeds in reducing viral loads in the blood to an undetectable level [1]. Indeed, 50% or more of HIV type 1 (HIV-1)-infected individuals receiving successful HAART suffer some level of HIV-1-associated neurocognitive disorder (HAND). Furthermore, most of the treatments for HAND remain ineffective [2]. The mechanisms of HIV infection causing neurocognitive impairment aren’t elucidated completely. The stained brain slides from acquired immunodeficiency syndrome (AIDS) patients showed a significant increase in amyloid deposition [3], which is closely related to the cognitive impairment of AIDS patients [4]. It has been shown that several mechanisms of vascular pathology caused by HIV-1 can be reproduced by the administration of the HIV-1 transactivator protein (Tat), which is made by HIV-1-infected cells [5]. HIV-1 Tat shows harmful effects on the development and progression of HAND. HIV-1 Tat is an inflammatory factor with neuroexcitability and neurotoxicity [6]; it can facilitate amyloid-beta (Aβ*)* accumulation by reducing Aβ degradation [7] and modulating Aβ transfer in vitro [8]. HIV-1 Tat causes increasing permeability of the blood–brain barrier (BBB) in vitro via downregulation of the expression of ZO-1, ZO-2, and occludin [7–9]. At the structural level, BBB dysfunction is associated with changes in tight junction (TJ) structure and functions [10], which are essential for maintaining low BBB permeability and integrity. TJ proteins include members of the claudin family, occludin, and ZO-1. Occludin is the first TJ protein to be found and is not essential for the formation of TJ chains, but the presence of occludin in the brain endothelial membrane is related to increased transepithelial electrical resistance (TEER) and decreased paracellular permeability [11]. Zonula occludens (ZO)-1 are other primary elements of TJ and they link other TJ proteins such as occludin to the actin cytoskeleton, which is essential for TJ formation [12]. Dysfunction of the BBB is related to neurocognitive deficits sustained in a variety of diseases such as stroke, Alzheimer’s disease (AD), and HIV-1 [13–15]. A functionally damaged BBB with a decreased clearance of Aβ from brain to blood could give rise to brain Aβ accumulation [16]. Therefore, inhibiting HIV-1 Tat-induced BBB dysfunction and Aβ accumulation can be a potential strategy for the treatment of HAND.

Aβ accumulation in the brain results in its gradual oligomerization [17]. Therefore, constant clearance of Aβ from the CNS into the blood is the most significant mechanism to prevent the potential neurotoxic accumulations of Aβ in the brain [18]. Aβ is cleared via enzyme-mediated breakdown or through non-proteolytic pathways. Neprilysin (NEP) is a rate-limiting Aβ-degrading enzyme that is highly expressed in human astrocytes and exhibits the strongest Aβ 1–40 and Aβ1–42 degradative activity [19]. The reduction of NEP levels may result in a significant decline in Aβ degradation, promoting Aβ deposition in the brain [20]. HIV-1 Tat-caused NEP dysfunction is detected in cerebral microvascular endothelial cells [7], neurons [3], and astrocytes [21]. Alternatively, Aβ may be transported across the BBB into the blood by the low-density lipoprotein receptor-related protein 1 (LRP1), while the transport of Aβ from the bloodstream into the brain is achieved by the receptor for advanced glycation end products (RAGE) [22]. Exposing hCMEC/D3 cells to HIV-1 or HIV-1 Tat result in markedly enhanced accumulation of intracellular Aβ due to increased RAGE expression [8, 23] and decreased LRP1 expression [8]. It is suggested that upregulation of NEP and LRP1 expression and downregulation of RAGE expression may become promising targets for the treatment of HAND.

Rho-kinase (ROCK), a serine/threonine kinase, is a key downstream effector of the small GTPase Rho; it alters the cytoskeleton to adjust cell migration and proliferation. It was reported that ROCK mediates BBB destruction [24], and activation of ROCK was observed to coincide with increased BBB permeability in the capillaries of AD mice [25]. ROCK1/ROCK2 inhibition ameliorates cognitive deficits, largely through decreased Aβ deposition, and promotes Aβ internalization in an AD model [26]. Thus, inhibiting ROCK might prevent cognitive decline. Previous studies from our group demonstrated that the Rho-kinase inhibitor hydroxyfasudil (HF) markedly restrained HIV-1 Tat-caused occludin downregulation, HIV-1 Tat-regulated LRP1, and RAGE expression in hCMEC/D3 [8]. Nonetheless, the underlying mechanism of whether HIV-1 Tat-caused BBB damage and Aβ accumulation in the mouse brain can be reversed by the ROCK inhibitor warrants further research. Moreover, ZO-1 and occludin express between adjacent cerebral microvessel endothelial cells [27]. The expression of NEP in the brain is relatively low, and it is mainly expressed in the middle membrane of cerebral cortex vessels and pyramidal neurons [28]. Previous studies had shown that the decrease of NEP expression in cerebral microvessels was negatively related to the deposition of Aβ around cerebral vessels [29]. However, LRP1 and RAGE are expressed in a variety of cells in the brain, while LRP1 and RAGE of cerebral microvessels are related to Aβ transport across the BBB [22]. Therefore, mice cerebral microvessels were used as experimental samples in this study. Our data indicate that ROCK inhibition by HF has protective effects on HIV-1 Tat-caused dysfunction of TJ and NEP/Aβ transfer receptor expression.

## Materials and methods

### Animals

C57BL/6 mice (22–25 g, 8 weeks old, male) were purchased from Guangxi Medical University Animal Center (Guangxi, China). The animals were housed under constant temperature (23 ± 1 °C), humidity (60 ± 10%) and light-controlled vivarium (12-h light/12-h dark cycle). Food and water were available adlibitum. The mice used in this study have been granted approval from the Committee on the Ethics of Animal Experiments of Guangxi Medical University (Certificate Numbers: SYXK 2014–0003) and the experiment had been proceeded in compliance with National Institutes of Health (NIH) Guide for the Care and Use of Laboratory Animals.

### Animal experiments

Recombinant HIV-1 Tat clade-B protein (amino acids 1 to 86, product#HIV-129) was obtained from Prospec (Rehovot, Israel). Previous research indicates that the dosage of HIV-1 Tat used in mouse experiments varied widely, especially in BBB damage and Aβ accumulation experiments [30, 31]. To identify what dosage of HIV-1 Tat would have a significant effect on BBB, animals were divided into 4 groups (*n* = 9 each) on a random basis and were administered with 0, 25, 50, and 100 μg/kg HIV-1 Tat via tail vein injection. The appropriate HIV-1 Tat concentration was obtained by detecting Evans blue (EB) leakage and EB fluorescence intensity by spectrophotometry and fluorescence microscopy, respectively, meanwhile by measuring the expression of ZO-1 of mouse brain microvessels by Western blotting analysis. The methods will be explained later.

Animals were randomly divided into 4 groups (*n* = 9 each): the control group, HIV-1 Tat group, HF group and HIV-1 Tat + HF group. In the control group, 1 h after intraperitoneal injection with 100 μl sterile saline, the mice were injected with 100 μl sterile saline via tail vein. In the HIV-1 Tat group, 1 h after intraperitoneal injection with 100 μl sterile saline, the mice were injected with 100 μl of HIV-1 Tat 100 μg/kg and sterile saline through the tail vein. In the HF group, 1 h after intraperitoneal injection with 100 μl of 10 mg/kg HF (product#ab145524, abcam, USA) and sterile saline, the mice were injected with 100 μl sterile saline through the tail vein. In the HIV-1 Tat + HF group, 1 h after intraperitoneal injection with 100 μl of 10 mg/kg HF and sterile saline, the mice were injected with 100 μl of HIV-1 Tat 100 μg/kg and sterile saline through the tail vein. The above treatment was daily for seven consecutive days. Significant side effects caused by HIV-1 Tat or HF treatment were not observed in the experiments. All mice were sacrificed 24 h after the last treatment and their brains were harvested.

### BBB permeability

The amount of EB leakage was detected as described earlier [32]. Twenty-two hours after the last treatment, mice of each group were given an EB (product#E2129, Sigma-Aldrich) solution (2%, 5 mL/kg) through tail vein injection. It was allowed to circulate for 2 h. Mice were anesthetized with sodium pentobarbital (1%, 30 mg/kg, i.p.) and were transcardially perfused with frozen sterile saline. The brains were quickly harvested; the cerebellum was removed, weighed, and homogenized lightly in 50% wt/vol trichloroacetic acid (product#T6399, Sigma-Aldrich). After processing with centrifugation at 14000 *g* for 15 min at 4 °C, the supernatant was carefully removed and the absorption of the supernatant at 620 nm was detected with a spectrophotometer (NanoDrop 2000, Thermo Fisher Scientific Inc.). The content of EB was valued as μg/g of brain tissue by a standard absorption curve.

After taking out the craniocerebral cover plate of mice, the brains were cut 2 mm forward and 2 mm backward of the anterior fontanelle with coronal incision according to the anatomical atlas of mouse stereotactic location compiled by George Paxinos. The brain tissues of frontal cortex were cut into 20-μm-thick slices through a cryostat (product#CM1850, Leica Microsystems, Wetzlar, Germany). Sections were stuck on gel-coated slices and triggered by blue light, then observed through a fluorescence microscope (product#BX-50, Olympus, Japan). The fluorescent intensity indicated the amount of EB leakage. The EB fluorescence intensity was detected by Image J software (National Institutes of Health, Bethesda, MD, USA).

### Western blotting analysis

Twenty-four hours after the last treatment, the mice were anesthetized with sodium pentobarbital (1%, 30 mg/kg, i.p.) and decapitated; then, their brains were removed. Mouse cerebral microvessels were isolated according to the research method of Hrvoje Brzica et al. [33]. The cerebral cortex (300 μg) was used to isolate the brain microvessels. Finally, the brain microvessels were suspended with 50ul brain microvascular buffer and stored at −20 °C for further use. To extract the total protein, the cerebral microvessels (25 ul) were homogenized in 100 ul lysis buffer (product#ab156034, Abcam, USA). Immunoblotting of ZO-1, occludin, NEP, LRP1 and RAGE were performed as described earlier [7, 8]. Rabbit anti-ZO-1 antibodies (product#ab96587, 1:1000, rabbit polyclonal; Abcam, Cambridge, UK), rabbit anti-occludin antibodies (product#13409–1-AP, 1:1000, rabbit polyclonal; Proteintech Group, Chicago, IL, USA), rabbit anti-NEP antibodies (product#ab256494, 1:500, rabbit polyclonal; Abcam), rabbit anti-RAGE antibodies (product#ab216329, 1:800, rabbit polyclonal; Abcam, Cambridge, UK), rabbit anti-LRP1 antibodies (product#ab92544, 1:20000, rabbit polyclonal; Abcam, Cambridge, UK), and rabbit anti-GAPDH antibodies (product#ab181602, 1:10000, rabbit polyclonal; Abcam) were used as the primary antibody. GAPDH was used as the loading control. Band density was measured by Image J software.

### RNA isolation and qRT-PCR

Cerebral microvessels were separated as mentioned before [33]. Total RNA was extracted from cerebral microvessels (25 ul). qRT-PCR of ZO-1, occludin, NEP, LRP1 and RAGE were performed as described earlier [7, 8]. The primer sequences were as follows: ZO-1 forward: 5′-GCCGCTAAGAGCACAGCAA-3′, ZO-1 reverse: 5′-TCCCCACTCTGAAAATGAGGA -3′, Occludin forward: 5′-TTGAAAGTCCACCTCCTTACAGA-3′, Occludin reverse: 5′-CCGG ATAAAAAGAGTACGCTGG-3′, NEP forward: 5′-CTCT CTGTGCTTGTCTTGCTC-3′, NEP reverse: 5′- GACGTTGCGTTTCAACCAGC-3′, RAGE forward: 5′- CTTGCTCTATGGGGAGCTGTA-3′, RAGE reverse: 5′- GGAGGATTTGA GC CACGCT-3′, LRP1 forward: 5′-ACTATGGATGCCCCTAAAACTTG-3′, LRP1 reverse: 5′- GCAATCTCTTTCACCGTCACA-3′, GAPDH forward: 5′- AGGTCGGTGTGAACGGAT TTG-3′, GAPDH reverse: 5′- TGTAGACCATGTAGTTGAGGTCA-3′, GAPDH was used to normalize the target gene mRNA levels, which were analyzed using the 2 –ΔΔCt method.

### Statistical analysis

SPSS 17.0 (Chicago, IL, USA) was used for statistical analysis. The data are presented as the means ± standard deviation. One-way Anova was used in conducting comparisons between groups; Student-Newman-Keuls (SNK) was used for the post hoc test. *P* < 0.05 was regarded to be significant.

## Results

### Dose-dependent effects of HIV-1 Tat on BBB permeability and ZO-1 protein levels

**Fig. 1:**
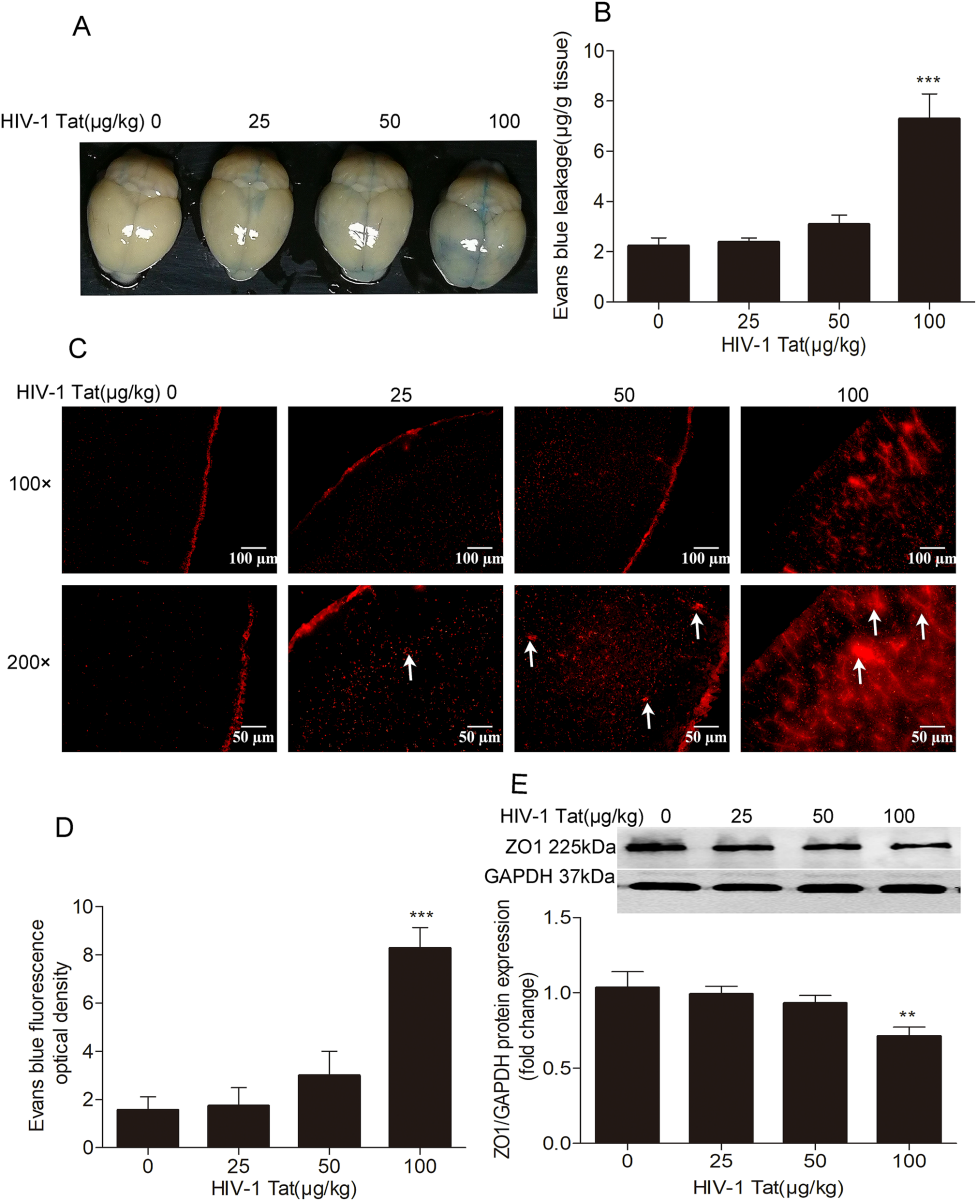
Effects of different concentrations of HIV-1 Tat on BBB permeability and ZO-1 protein expression. (**a**): With the dose of HIV-1 Tat growing, the color of the brain surface gradually became blue. The amount of EB leakage was determined by the tissue homogenate method (**b**) and fluorescence microscopy in the frozen tissue section (**c**–**d**). The arrows represent EB exudation. Compared to the control group, the amount of EB leakage and EB fluorescence intensity also increased in the HIV-1 Tat 25 μg/kg group and HIV-1 Tat 50 μg/kg group, but the difference was not statistically significant in comparison with the control group (*P* > 0.05). The amount of EB leakage and EB fluorescence intensity significantly increased in the HIV-1 Tat 100 μg/kg group in comparison with the control group (***P < 0.001). HIV-1 Tat increased the amount of EB leakage in a dosage-dependent manner. E: Western blotting was used to determine the protein level of ZO-1 in mouse brain microvessels. HIV-1 Tat decreased the protein level of ZO-1 in a dosage-dependent manner. Compared to the control group, the protein level of ZO-1 protein significantly reduced in HIV-1 Tat 100 μg/kg group (**P < 0.01). The data represent the mean ± standard deviation of the three independent experiments

EB binds to serum albumin, which prevents it from crossing the normal BBB, but dysfunctional BBB allows EB to pass through. Hence, EB leakage is used to evaluate BBB function [34]. HIV-1 Tat has been considered to destroy BBB in vitro [7, 8, 35] and in vivo [31]. To assess the permeability of BBB, EB leakage and EB fluorescence intensity were examined by spectrophotometry and fluorescence microscopy, respectively, and the ZO-1 expression in mouse brain microvessels was assessed with Western blotting. With the dose of HIV-1 Tat increasing, the mouse brain in blue (Fig. 1a), the amount of EB leakage (Fig. 1b), the mouse brain fluorescence signal (Fig. 1c) and the EB fluorescence intensity (Fig. 1d) were gradually enhanced; however, the protein levels of ZO-1 (Fig. 1e) were gradually reduced. HIV-1 Tat at 100 μg/kg body weight significantly increased EB leakage and EB fluorescence intensity compared to the control group (***P < 0.001) and reduced the expression of ZO-1 compared to the control group(**P < 0.01).

### Role of the Rho/ROCK signaling pathway in HIV-1 Tat-induced changes in BBB permeability

**Fig. 2:**
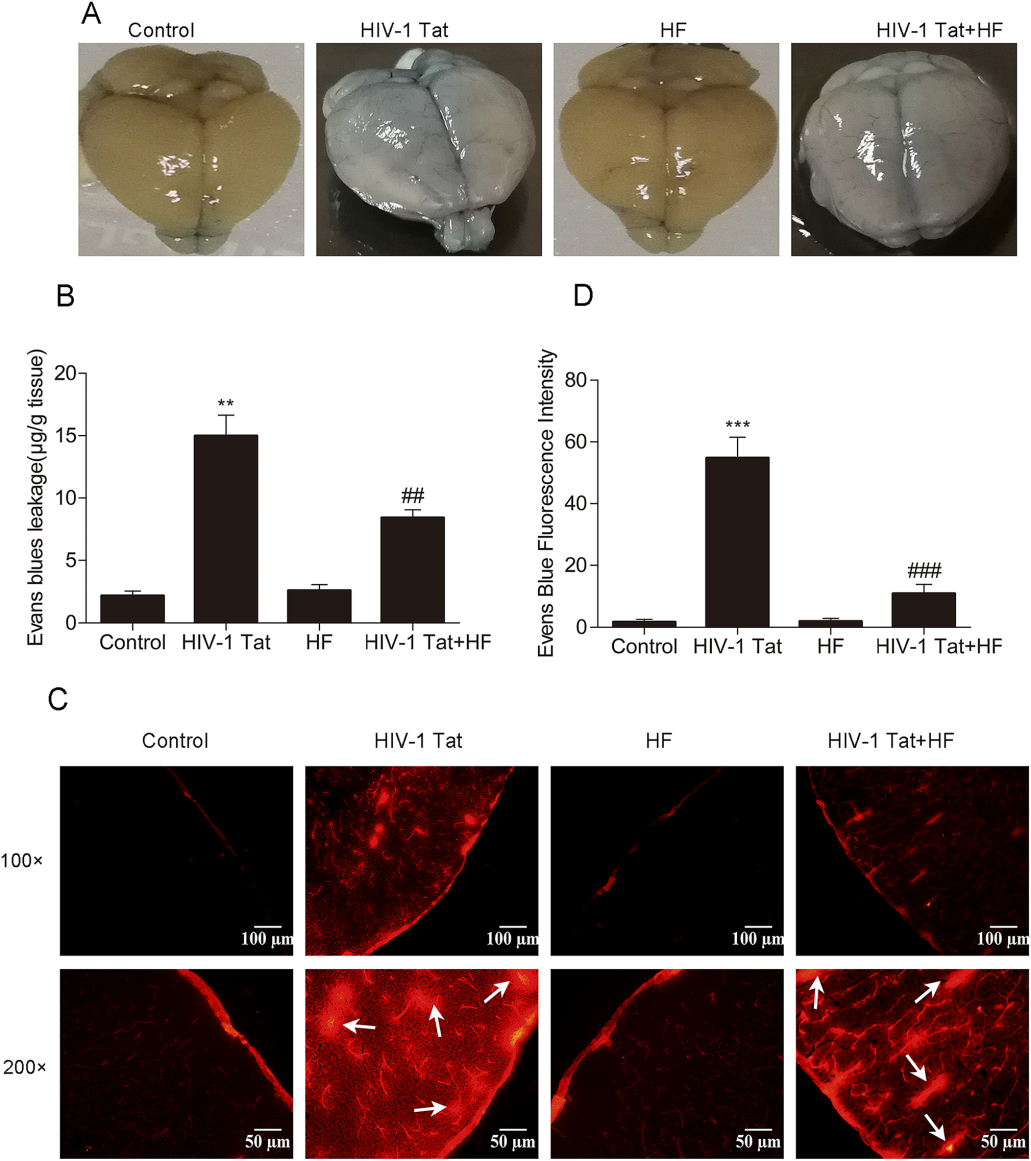
The permeability of the blood–brain barrier to EB was determined by the tissue homogenate method and fluorescence microscopy in the frozen tissue section. (**a**): Gross specimen: EB staining on the mouse brain surface. (**b**): The amount of EB was determined by the tissue homogenate method. (**c**) and (**d**): The exudation amount of EB was determined by fluorescence microscopy in the frozen tissue section. The arrows represent EB exudation. Compared to the control group, the mouse brains were stained in blue; the exudation amount of EB, EB fluorescence signal, and intensity significantly increased in HIV-1 Tat 100 μg/kg group (***P < 0.001, **P < 0.05). Compared to HIV-1 Tat 100 μg/kg group, the blue staining of the mouse brain surface became shallow; the EB leakage amount, EB fluorescence signal, and intensity decreased significantly in the HIV-1 Tat 100 μg/kg + HF group (### P < 0.001, ##P < 0.01). The data represent the mean ± standard deviation of the three independent experiments

The effect of HF on HIV-1 Tat-caused BBB damage was evaluated by EB leakage and frozen slices of brain tissues. When 100 μg/kg of HIV-1 Tat was given to the C57BL/6 mouse once a day for seven consecutive days, the mouse brain of the HIV-1 Tat group was clearly stained in blue in the cerebral cortex surface (Fig. 2a). In addition, the EB leakage of the HIV-1 Tat group (Fig. 2b) showed a significant increase compared to the control group and HF group (**P < 0.01). However, in the HF + HIV-1 Tat group, the blue staining in the mouse brain (Fig. 2a) was not conspicuous and EB leakage (Fig. 2b) significantly decreased in comparison to the HIV-1 Tat group (##P < 0.01). Moreover, the mouse brain EB fluorescent signals and fluorescence intensity of HIV-1 Tat group significantly increased in comparison to the control group (Fig. 2c–d) (***P < 0.001). However, the EB fluorescent signals in the mouse brain and its fluorescence intensity went down in the HF + HIV-1 Tat group compared to the HIV-1 Tat group (###P < 0.001). The blue staining in the mouse brain, the amount of EB leakage, the mouse brain EB fluorescent signals, and fluorescence intensity showed no significant difference between the HF group and the control group.

### Role of the Rho/ROCK signaling pathway in HIV-1 Tat-induced changes in ZO-1 and occludin of mouse brain microvessels

**Fig. 3:**
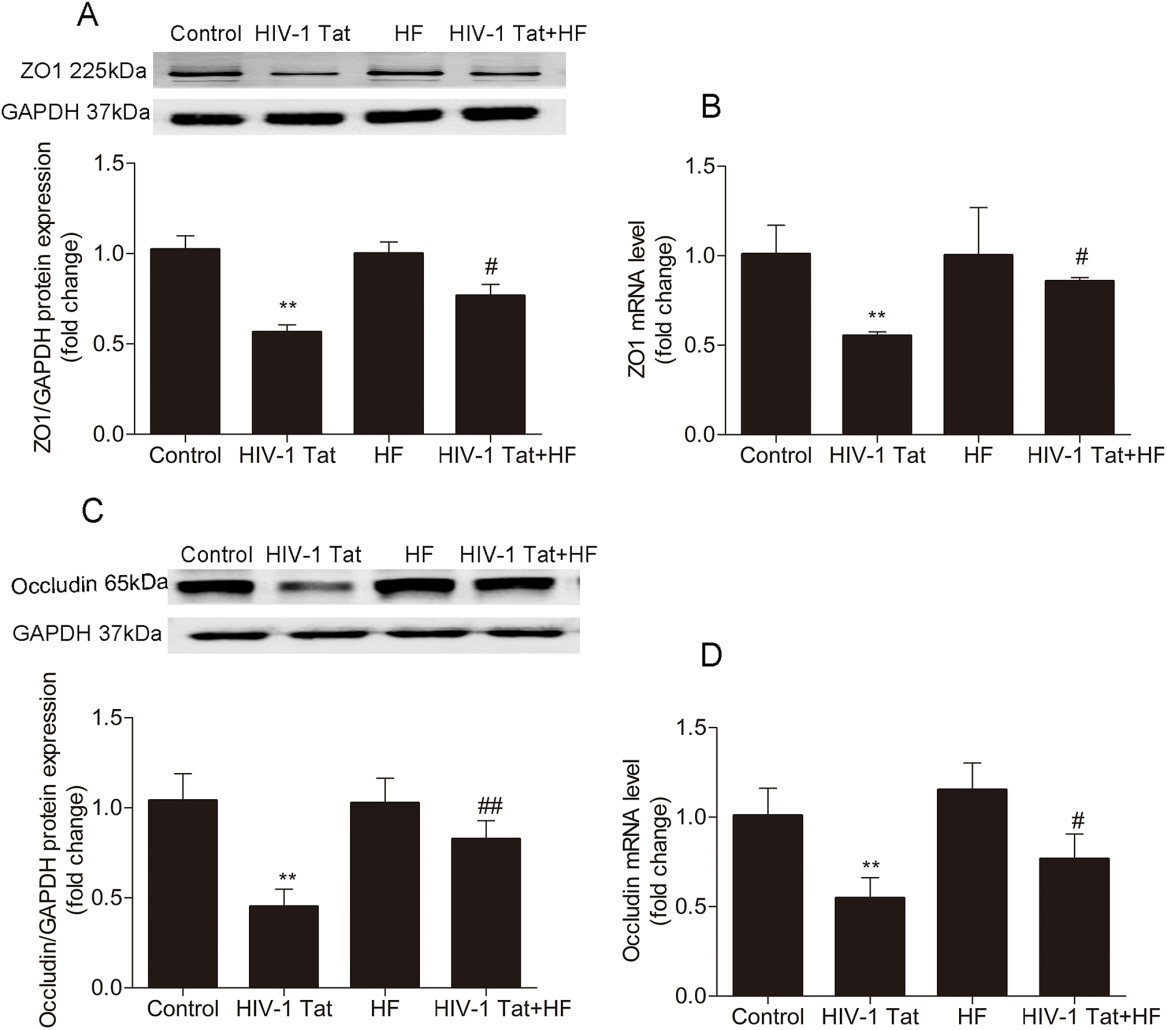
The Rho/ROCK signaling pathway involved in HIV-1 Tat-induced changes in ZO-1 and occludin expression of mouse brain microvessels. The protein and mRNA levels of ZO-1 and occludin in mouse cerebral microvessels were detected by Western blotting and qRT-PCR, respectively. The protein levels of ZO-1 and occludin were shown in (**a**) and (**c**). Their mRNA levels were shown in (**b**) and (**d**). Compared to the control group, HIV-1 Tat (100 μg/kg) downregulated the protein and mRNA levels of ZO-1 and occludin (** P < 0.01). Compared to the HIV-1 Tat 100 μg/kg group, HF pretreatment significantly upregulated the protein and mRNA levels of ZO-1 and occludin (#P < 0.05, ##P < 0.01). These data represent the mean ± standard deviation of the three independent experiments

HIV-1 Tat caused changes in TJ protein expression through the Ras signaling pathway in vitro [9] . To address the effect of ROCK on HIV-1 Tat-caused changes of ZO-1 and occludin protein in vivo, the ROCK-specific inhibitor HF was administered 30 min before being applied to HIV-1 Tat once a day for seven consecutive days. The protein levels and mRNA levels of ZO-1 and occludin in mouse brain microvessels were detected by Western blotting and qRT-PCR, respectively. ZO-1 and occludin protein levels as shown in Fig. 3a and c and their mRNA levels as shown in Fig. 3b and d were downregulated following HIV-1 Tat exposure (**P < 0.01 vs the control group in both Western blotting and qRT-PCR) but were significantly upregulated in the HF + HIV-1 Tat group (#*P* < 0.05 or ##P < 0.01 vs the HIV-1 Tat group in both Western blotting and qRT-PCR).

### Role of the Rho/ROCK signaling pathway in HIV-1 Tat-induced changes in NEP, RAGE and LRP1 in mouse brain microvessels

**Fig. 4:**
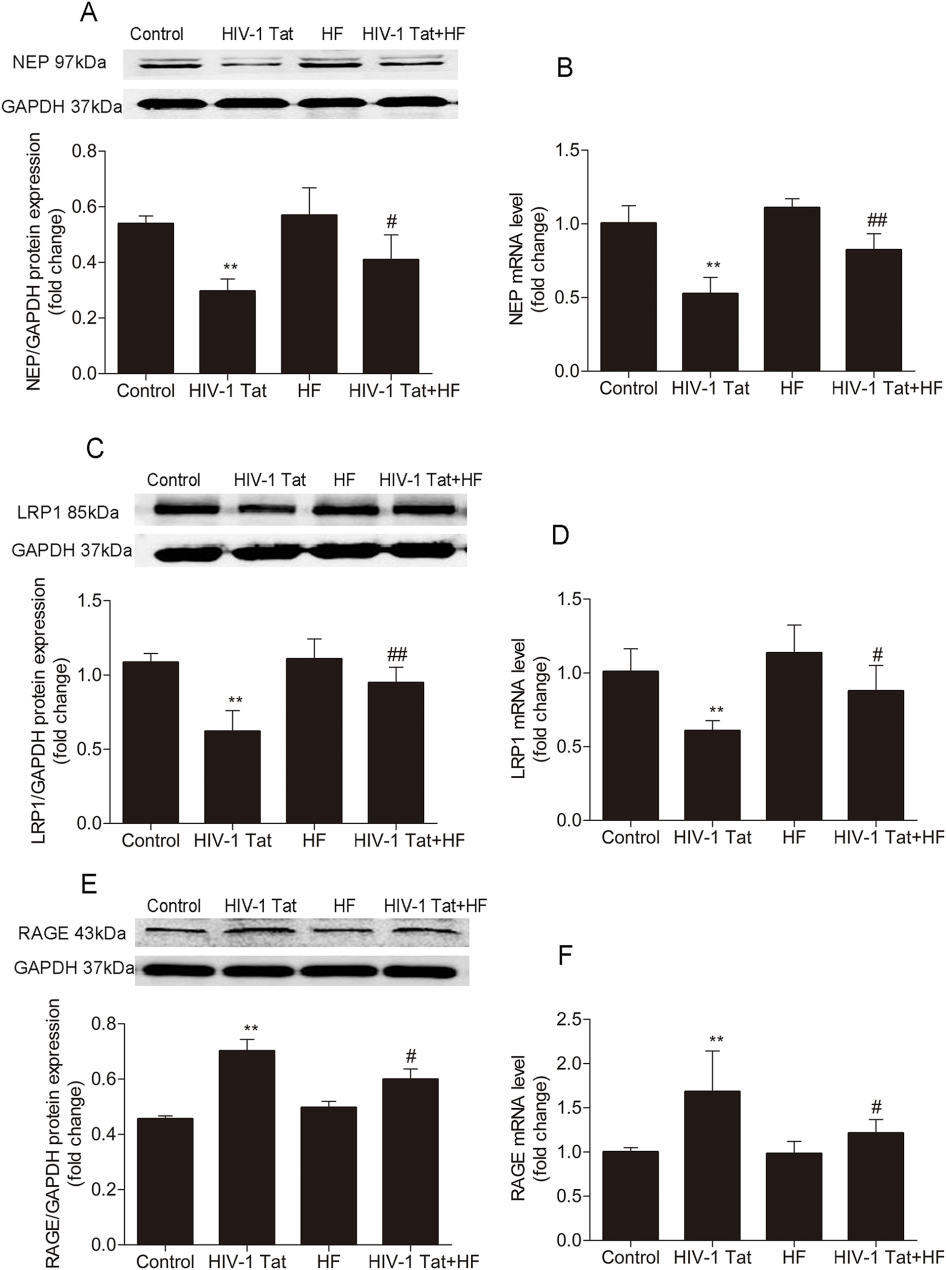
The Rho/ROCK signaling pathway involved in HIV-1 Tat-induced changes of NEP, LRP1 and RAGE of mouse brain microvessels. The protein levels and mRNA levels of NEP, LRP1, and RAGE in the mouse brain microvessels were detected by Western blotting and qRT-PCR, respectively. NEP and LRP1 protein levels (**a** and **c**) as well as mRNA levels (**b** and **d**) declined following HIV-1 Tat (100 μg/kg) exposure (compared to the control group, **P < 0.01 in both Western blotting and qRT-PCR), but were upregulated in the HIV-1 Tat 100 μg/kg + HF group (##P < 0.01 or #P < 0.05 vs the HIV-1 Tat 100 μg/kg group in both Western blotting and qRT-PCR). RAGE protein and mRNA levels (**e**, **f**) were ascended after HIV-1 Tat (100 μg/kg) exposure in comparison with the control group (**P < 0.01 in both Western blotting and RT-PCR), but were downregulated in the HIV-1 Tat 100 μg/kg + HF group (#P < 0.05 vs the HIV-1 Tat 100 μg/kg group in both Western blotting and qRT-PCR). Data are representative of three independent experiments and shown as the means ± standard error of the mean

Our previous study had showed that HF significantly inhibited HIV-1 Tat-regulated Aβ transferring protein LRP1 and RAGE expression in hCMEC/D3 cells [36]. To determine the underlying mechanism of the effect of ROCK on HIV-1 Tat-induced dysfunction of NEP/Aβ transfer receptor expression in the mouse brain, HF was administered 30 min before giving HIV-1 Tat once a day for seven consecutive days. The protein and mRNA levels of NEP, LRP1 and RAGE in mouse brain microvessels were measured by Western blotting and qRT-PCR, respectively. NEP and LRP1 protein levels (Fig. 4a and c, respectively), along with RNA levels (Fig. 4b and d) declined after HIV-1 Tat administeration in comparison with the control group (**P < 0.01 in both Western blotting and qRT-PCR). However, they were upregulated in the HF + HIV-1 Tat group (##P < 0.01 or #*P* < 0.05 vs the HIV-1 Tat group in both Western blotting and qRT-PCR). The protein and mRNA levels of RAGE (Fig. 4e–f) were ascended after HIV-1 Tat exposure in comparison with the control group (**P < 0.01 in both Western blotting and RT-PCR), but were downregulated in the HF + HIV-1 Tat group (#P < 0.05 vs the HIV-1 Tat group in both Western blotting and qRT-PCR).

## Discussion

BBB dysfunction is always accompanied by severe neurological dysfunction and neurodegeneration, including HAND [37]. Our previous data, along with other groups’ data, revealed that exposure of cerebral endothelial cells to HIV-1 Tat caused the expression of ZO-1 [7] and occludin [8] to be downregulated; BBB permeability and Aβ accumulation were upregulated [7]. In this experiment, HIV-1 Tat affected BBB permeability and the expression of ZO-1 in a dosage-dependent manner in the mouse brain; the effect of HIV-1 Tat 100 μg/kg was the most significant (Fig. 1). Therefore, HIV-1 Tat 100 μg/kg was selected for use in the rest of the experiment. The dosage in the current study is different from the findings obtained in previous studies due to the differences in HIV-1 Tat peptide length and purity, as well as differences in method and duration of administration. In the research of William A. banks et al., it was found that HIV-1 Tat passes through the BBB via a unsaturable mechanism with a unidirectional influx rate of about 0.490 Al/g/min. About 0.126% of an intravenous dose of HIV-1 Tat entered each g of brain [38]. It is consistent with the fact that only a small fraction of intravenous administered Tat can enter the brain of mice [5].

ROCK1/ROCK2 inhibitors rescued the efficacy of BBB damage in several CNS diseases, including experimental autoimmune encephalomyelitis, cerebral ischemia, and intracerebral hemorrhage [39]. Activation of Rho-A/ROCK changed TJ and F actin cytoskeleton organization, then upregulated epithelial TJ permeability [26]. HIV-1 Tat caused the disruption of TJ proteins by activating the Ras/mitogen-activated protein kinase (MAPK) pathway in brain endothelial cells [9]. Upstream of the MAPK signaling pathway, the Rho/ROCK signaling pathway may be involved in HIV-1 Tat-induced BBB dysfunction. In this experiment, the levels of Rho-A or ROCK protein were examined by Western blotting, but their total amount did not change. It was also found that the levels of Rho-A or ROCK did not change after exposure to HIV-1 Tat, but their activity was affected in the endothelial cells of the BBB [40]. Our previous studies in vitro showed that the Rho-kinase inhibitor HF markedly restrained HIV-1 Tat-caused occludin downregulation [8] and HIV-1 Tat inhibited the expression of ZO-1 in brain endothelial cells via the Ras signaling pathway [7]. In this in vivo study, the Rho-kinase inhibitor HF reversed the HIV-1 Tat-caused downregulation of the protein and mRNA levels of ZO-1 and occluding; it also reversed upregulation of EB leakage and EB fluorescence intensity in the mouse brain (Figs. 2 and 3). In the study of Chen et al., after administrated with HF 12 h, the mice were injected with 25 μg HIV-1 Tat via the internal carotid artery. HIV-1 Tat also reduced the expression of ZO-1 and claudin 5 in the hippocampus of mice; this effect could be reversed by HF [31]. The similar results can be observed in this study, even though the way of interventions and experimental specimens were different. Collectively, these findings indicate that HF protected against HIV-1 Tat-induced BBB dysfunction partly by inhibiting the Rho/ROCK signaling pathway in vivo.

Perivascular Aβ accumulation increased in brains with HIV-1 encephalitis (HIVE)- and HIV-1-associated dementia [4]. In cell-based studies, exposure to HIV-1 Tat significantly enhanced the extracellular levels of Aβ [3, 7]. After HIV-1 infection, several mechanisms contributed to upregulating the levels of Aβ, such as the increase of its production [41], deficient enzymatic degradation [3], and an increased transport mechanism across the BBB [23]. In the brain of an HIV infected patient, Aβ accumulation and deposition drove the pathogenic cascades of neurological disorders; this contributes toward aging or associated dementias [41]. Therefore, targeting Aβ degradation and transport may be a potential strategy for the treatment of HAND. Growing evidence suggests that ROCK influence Aβ production and clearance. In vitro, ROCK2 has been shown to regulate phosphorylation of APP at threonine 654 site, which is critical for b-secretase cleavage [42]. On the other hand, ROCK can also influence Aβ production through regulating APP intracellular transport and Aβ secretion [43]. ROCK1 is also thought to be the promoter of Aβ autophagy; the overexpression of ROCK1 promoted internalization of Aβ and formation of autophagosomes in vitro [44]. ROCK inhibition reduced the brain’s amyloid protein level in a transgenic mouse model of AD [45]. These findings show a strong link between ROCK and Aβ clearance.

NEP is a major Aβ-degrading enzyme, showing the most potent degrading activity of Aβ 1–40 and Aβ 1–42 [19]. A negative correlation between NEP and Aβ plaque load was repeatedly confirmed in post-mortem human brains [46] and in animal models [19]. In an in vitro assay, HIV-1 Tat inhibited 80% of NEP. Exposure of HIV-1 Tat to brain cultures showed an increase of soluble Aβ [3]. Previously in our study [7], HIV-1 Tat diminished NEP expression and weakened NEP immunoreactivity in hCMEC/D3 cells; this effect was reversed by the Ras signaling pathway inhibitor farnesylthiosalicylic acid. In keeping with earlier findings [21], treatment with HIV-1 Tat in the mouse reduced expression of NEP protein and mRNA levels in this study; this effect may directly result in reducing Aβ degradation in the brain which, in turn, added to Aβ accumulation, but inhibition of ROCK by HF effectively restrained HIV-1 Tat-caused NEP reduction in the mouse (Fig. 4a–b). However, cellular NEP expression levels were not downregulated in HIV-1pYu2 infection of human monocyte-derived macrophages [47]. The expression and activity of NEP were reduced by exposing brain vascular endothelial cells or glial cells to HIV-1 Tat [7, 21]. NEP protein expression positively correlates with enzymatic activity [3]. These different results may be attributed to different HIV-1 subtypes and cell types. NEP double bands are shown in Fig. 4a. This is likely to be a phosphorylation or methylation of NEP which is lost upon HIV-1 Tat treatment and is reverted after ROCK inhibition via HF. Similar NEP double bands have been found in other studies [48], but no further studies have been carried out yet.

A functionally impaired BBB with a decreased brain-to-blood clearance of Aβ could facilitate Aβ accumulation [16]. If the BBB is impaired, the circulating Aβ can also enter the brain through pro-inflammatory cytokines, possibly caused by the Aβ-RAGE interaction [49]. Peptides such as Aβ cannot directly cross the BBB, but they can be transferred via the specific transferring receptors LRP1 and RAGE. RAGE transports Aβ from the bloodstream into the brain, while LRP1 undertakes the opposite. LRP1 and RAGE regulate Aβ levels in the brain by transporting Aβ across the BBB. The expression of microvascular LRP1 decreased, while the level and immune activity of RAGE increased in the human brain of AD [22]. RAGE is a subtype of Caveolae and is expressed at relatively low levels at the BBB. Our previous data revealed that silencing the Cav-1 gene greatly prevented HIV-1 Tat-caused upregulation of RAGE and downregulation of LRP1 [35]. Ibolya E András and colleagues in 2010 reported that simvastatin weaken HIV-1-induced upregulation of RAGE expression; however, HIV-1 did not affect LRP1 expression in brain endothelial cells [23]. In this study, treatment with HIV-1 Tat gave rise to downregulation of LRP1 protein and mRNA levels, as well as upregulation of RAGE protein and mRNA levels in the mouse brain microvessels. This finding may lead directly to Aβ accumulation in the brain. However, this effect was reversed by HF (Fig. 4c–f). It is consistent with our previous in vitro study [8]. These data indicate that restraining ROCK may weaken HIV-1 Tat-caused dysfunction of Aβ transendothelial transfer and degradation in vivo.

In summary, inhibition of Rho/ROCK signaling by HF in the mouse has a strong protective effect on HIV-1 Tat-induced downregulation of ZO-1, occludin, NEP, and LRP1, as well as upregulation of RAGE and BBB permeability. This may lead to the degradation and transport dysfunction of Aβ in the brain. These findings suggest that Rho/ROCK signaling pathway is a crucial element in the HIV-1 Tat-induced BBB destruction and dysfunction of NEP/Aβ transfer receptor expression. Targeting the Rho/ROCK signaling pathway may be a promising option to treat HAND.

## Data Availability

All data generated or analyzed during this study are included in this article.
